# Epidemiology of diabetic foot disease and diabetes-related lower-extremity amputation in Australia: a systematic review protocol

**DOI:** 10.1186/s13643-017-0488-5

**Published:** 2017-05-18

**Authors:** Jaap J. van Netten, Mendel Baba, Peter A. Lazzarini

**Affiliations:** 1 School of Clinical Sciences, Queensland University of Technology, Brisbane, QLD Australia; 2 Diabetic Foot Australia, West End, QLD 4101 Australia; 3 Wound Management Innovation Cooperative Research Centre, Brisbane, QLD Australia; 4 Department of Podiatry, Metro North Hospital & Health Service, Queensland Health, Brisbane, QLD Australia; 5 Independent Consultant, Perth, Australia

**Keywords:** Diabetes mellitus, Diabetic foot disease, Foot ulcer, Amputation, Epidemiology, Incidence, Prevalence, Australia

## Abstract

**Background:**

Diabetic foot disease is associated with major morbidity, mortality, costs, and reduction of a person’s quality of life. Investigating the epidemiology of diabetic foot disease is the backbone of diabetic foot research and clinical practice, yet the full burden of diabetic foot disease in Australia is unknown. This study aims to describe the protocol for a systematic review of the epidemiology of diabetic foot disease and diabetes-related lower-extremity amputation in Australia.

**Methods—search:**

The systematic review will be performed according to the Preferred Reporting Items for Systematic Review and Meta-Analyses guidelines. PubMed and EMBASE will be searched for publications in any language and without restrictions to date. Two independent investigators will screen publications for eligibility, with publications reporting Australian population-based incidence or prevalence of diabetic foot disease or diabetes-related lower-extremity amputation to be included. Additionally, a forward literature search will be performed in Google Scholar, and a grey literature search will be performed to identify government publications.

**Methods—assessment:**

Quality assessment will be performed using customised checklists. The summary statistic used for each study will be an incidence or prevalence proportion of diabetic foot disease or diabetes-related lower-extremity amputation. The standard error for each proportion will be calculated. A meta-analysis will be performed when three or more publications of adequate quality, reporting on similar outcomes and in similar populations, are identified.

**Discussion:**

The results of this systematic review can be used to adequately inform stakeholders in the field of diabetic foot disease on the extent of the problem in incidence and prevalence of diabetic foot disease in Australia, and to help guide appropriate use of resources to reduce the burden of this disease.

**Systematic review registration:**

PROSPERO CRD42016050740

**Electronic supplementary material:**

The online version of this article (doi:10.1186/s13643-017-0488-5) contains supplementary material, which is available to authorized users.

## Background

Diabetic foot disease is a complication of diabetes associated with major morbidity, mortality, costs, and reduced quality of life [1–4]. Diabetic foot disease typically presents as ulcers, infection, and Charcot foot in the presence of peripheral neuropathy or peripheral arterial disease in people with diabetes [5], and is the most important precursor for lower-extremity amputations [6]. Global prevalence of diabetic foot disease is estimated around 6% [7], whilst diabetes-related lower-extremity amputation incidence shows great global variability [8].

Investigating the epidemiology of a disease is the backbone of both research and clinical practice. Quantification of the problem is required to direct service provision, enable efforts for improvement, benchmark disease outcomes, and act as baseline to measure change. Australia has been reported to have one of the lowest rates of diabetic foot disease [7], yet one of the highest rates of diabetes-related amputation [9, 10]. However, these rates have never been systematically investigated. Therefore, the full burden of diabetic foot disease in Australia is unknown.

Whilst Australia is similar to other developed nations with regard to its ageing and urban population [11], it has many unique characteristics to other nations. For instance, Australia’s rural population is spread over much larger geographical distances and therefore access to health system services can prove more difficult compared to other developed nations. Furthermore, the indigenous population with diabetes in Australia has been reported to be 38 times more likely to undergo amputation than the non-indigenous (predominantly Caucasian) population [12]. These factors make Australia relatively unique compared to other developed nations, particularly European nations with much smaller geographical differences, and with the non-Caucasian populations presenting with lower risks of amputation compared to the Caucasian population [13].

All these factors warrant the need for a study investigating the burden of this disease in Australia. This study aims to systematically review the epidemiology of diabetic foot disease and diabetes-related lower-extremity amputation in Australia.

## Methods

The systematic review will be performed according to the Preferred Reporting Items for Systematic Review and Meta-Analyses (PRISMA) guidelines, with this protocol written according to the PRISMA-P checklist (see Additional file 1: Appendix 1) [14, 15]. The systematic review has been prospectively registered in the PROSPERO database for systematic reviews (CRD42016050740). Protocol amendments will be documented in PROSPERO.

### Search strategy

The following databases will be searched: PubMed and Excerpta Medica Database (EMBASE) via Ovid SP and will include publications in any language and without restrictions in date. Before performing the search, a validation set of approximately 10 publications was created and tested. Each publication in the set needed to be identified in the search. The validation set was created by including key publications known to the authors that fit the scope of this systematic review. The final search string is shown in Table 1.

**Table 1:** Search strings for PubMed and EMBASE

	PubMed		EMBASE
*#*	*Diabetic foot disease string*	#	*Diabetic foot disease string*
1	(“Ulcer”[Mesh] OR ulcer*[tw])	1	‘ulcer’/exp
2	(“Foot”[Mesh] OR foot[tw] OR feet[tw])	2	ulcer:ab,ti
3	“Foot Ulcer”[Mesh]	3	#1 OR #2
4	(“Foot Diseases”[Mesh] OR foot problem*[tw] OR foot disease*[tw])	4	‘foot’/exp
5	diabet*[tw]	5	(foot or feet):ab,ti
6	#1 AND #2 AND #5	6	#4 OR #5
7	#3 AND #5	7	#3 AND #6
8	#4 AND #5	8	‘foot ulcer’/exp
9	diabetic foot[tw]	9	‘foot disease’/exp
10	diabetic feet[tw]	10	(foot disease* or foot problem*):ab,ti
11	#6 OR #7 OR #8 OR #9 OR #10	11	diabet*:ab,ti
		12	#7 AND #11
		13	#8 AND #11
		14	#9 AND #11
		15	#10 AND #11
		16	‘diabetic foot’/exp
		17	#12 OR #13 OR #14 OR #15 OR #16
	*Amputation string*		*Amputation string*
12	Amput* [tw]	18	‘amput*’/exp
13	Diabet* [tw]	19	diabet*:ab,ti
14	#12 AND #13	20	#18 AND #19
	*Outcomes string*		*Outcomes string*
15	Incidence	21	Incidence/exp
16	Prevalence	22	Prevalence/exp
17	(#15 OR #16)	23	#21 OR #22
18	Austral*	24	Austral*/exp
19	#17 AND #18	25	#23 AND #24
	*Final search*		*Final search*
20	#11 OR #14	26	#17 OR #20
21	#19 AND #20	27	#25 AND #26

### Data management

Preliminary searches resulted in 155 records and 20 potentially eligible publications. Records and data can therefore be managed using Microsoft Excel (Microsoft Corporation, Redmond, WA, USA). All records will be imported from PubMed and EMBASE into an Excel file. This file will be used for study selection. Keeping the master copy and all management of this file is the responsibility of the first author. The Statistical Package for the Social Sciences (IBM Corporation, Armonk, NY, USA) will be used for data analysis, with data imported by the first author.

### Study selection

Title and abstracts will be screened by two independent investigators (JJvN and MB) for inclusion in the full-text assessment. Criterion for inclusion for full-text assessment is any mention of epidemiological data on diabetic foot disease or lower-extremity amputation in an Australian setting. A publication identified for inclusion by either one of the investigators will be included for full-text assessment. Cohen’s kappa will be calculated for screening agreement by the two investigators [16].

Included full-text publications will be assessed by two independent investigators (JJvN and MB) for eligibility using the inclusion and exclusion criteria. Inclusion criteria will be based on publications reporting Australian population-based incidence or prevalence of diabetic foot disease or diabetes-related lower-extremity amputation. Exclusion criteria will be based on publications reporting data from a single centre or selected centres (unless general- or diabetes-population information for the selected centres is provided or if data is adjusted or compared to the overall Australian population demographics); publications reporting lower-extremity amputation incidence or prevalence without differentiating between people with and without diabetes; and publications not reporting original data. No restrictions are placed on study duration, study period, or date of publication. Disagreements between investigators will be resolved with discussion until consensus is reached, with a third investigator to be involved if needed.

Diabetic foot disease will be defined as “infection, ulceration or destruction of tissues of the foot associated with neuropathy and/or peripheral artery disease in the lower extremity of people with diabetes” [17]. Diabetes-related lower-extremity amputation will be defined as “resection of a segment of the lower-extremity through a bone or joint” in a person with diabetes [17]. This will be further differentiated as major amputation (any resection proximal of the ankle) and minor amputation (any resection through or distal of the articulation of the ankle) [17]. However, we are mindful that different definitions may have been used in different studies and we will also include (and report) other definitions if used.

The denominator population will be defined as the general or diabetes population reported for a specified geographical area, with key characteristics (age and gender) to be reported for this population in the publication. Publications reporting in-hospital epidemiology of diabetic foot disease or diabetes-related lower-extremity amputation will be included, when data from the complete hospital population is given, and provided more than one hospital is included in the publication. Common risk factors for diabetic foot disease may also be used to further specify the “at-risk population” in the denominator (when available in a publication), and these include peripheral neuropathy, peripheral artery disease, and foot deformities [17, 18].

After completion of selection of the final included publications, a forward literature search will be performed via Google Scholar (https://scholar.google.com.au). This may help to identify early online publications that have not been indexed (since Google Scholar does not rely on indexation before publications are included in their database), and it may capture some grey literature as well (such as dissertations and conference proceedings). One author will assess the publications found in the forward literature search. All publications identified for inclusion by this author will be screened for eligibility as per the above study selection procedure of full-text publications.

### Grey literature search

As the authors are aware of government publications on the primary outcomes of this systematic review, a search to identify those publications and other grey literature meeting the inclusion and exclusion criteria will be undertaken. This search will consist of the following steps, performed by one author:

-Identifying and downloading the known Australian state and federal government publications meeting the inclusion and exclusion criteria
-Typing the title of the government publications in an incognito window of Google (www.google.com.au) and screening the first 10 pages of results by one investigator
-Contacting the author of the government publications to inquire about any further reports, either following the publication found or additional publications
-Contacting five external Australian content experts in the field to ask for known grey literature reporting diabetic foot disease epidemiology

As both EMBASE and Google Scholar include conference presentations and dissertations, the searches in those databases described elsewhere in this manuscript can also be regarded as covering part of the grey literature search.

All publications identified for inclusion in the grey literature search will be screened for eligibility as per the above study selection procedure of full-text publications.

### Data collection

Data from each publication will be extracted by one investigator, after which a second investigator will check the extracted data. Investigators will not extract data from an article of which they were (co-)author to prevent potential bias or conflict of interest. The primary outcome is the incidence or prevalence of diabetic foot disease and diabetes-related lower-extremity amputation. Data extracted will include setting, period, study design, use of prevalence or incidence, number of general or diabetes population, key characteristics (age, sex, foot disease risk factors, other) within the population, definition of diabetic foot disease or amputation, classification of diabetic foot disease or amputation level, other characteristics of diabetic foot disease or amputation, and if the diabetic foot disease or amputation is the first ever incidence or a recurrence. Authors will be contacted if data to be extracted are unclear from the original publication.

### Quality assessment

As diabetic foot disease and amputation pose specific requirements on study reporting, no specific quality assessment instrument is suited to the data and research aim of this review. Therefore, a customised approach will be followed. In line with conclusions from a recent systematic review on tools for assessing the quality and susceptibility to bias in observational studies, a transparent checklist approach has been chosen, concentrating on the few—principal—potential sources of bias identified from this systematic review [19]. The customised checklist developed to assess selection and information bias by the investigators is based on checklists used by other systematic reviews on population-based studies investigating mortality after amputation [20] and diabetic foot disease [21], which in turn were based broadly on the Meta-analysis Of Observational Studies in Epidemiology guidelines [22] and the Newcastle-Ottawa scale [23]. Our checklist aims to assess selection and information bias.

Five criteria will be assessed for each included publication by two independent investigators (Table 2). Item C is outcome specific and is scored twice when both primary outcomes are included. Each criterion is scored with a “+”, “?”, or “−”. The criteria chosen are based primarily on the quality of the reporting of the geographical area, diabetic foot disease or amputation numerator, population denominator, information flow, and study design. These criteria are all known to be important aspects of bias in diabetic foot disease research [21, 24]. Disagreements will be resolved by discussion until consensus is reached, with a third investigator to be involved if consensus is unable to be reached. Investigators will not assess the quality of an article of which they were (co-)author to prevent any conflict of interest.

**Table 2:** Customised checklist for risk of bias assessment

A	Quality of description of included geographical area or data sources	
+	Clear description of geographical area and participating centres within the area or data sources. For example, data are presented for the number, sex, and age distribution of the population. Where relevant, providing complete referencing details to original data collection procedures or sources is adequate	
?	Unclear or incomplete description of geographical area, its residents, and hospitals or data sources used	
−	No description of investigated area	
B	Quality of description of included population denominator	
+	Clear description of inclusion and exclusion criteria is presented. Minimal criteria were considered sex, age, and (definition of) diabetes	
?	Unclear or incomplete description of inclusion and exclusion criteria	
−	No description of inclusion and exclusion criteria	
C	Quality of description of diabetic foot disease or amputation numerator	
	Diabetic foot disease	Amputation
+	Clear description of reported diabetic foot disease characteristics in line with a published classification system using objective measurement methods	Clear description of the levels of amputation in line with international guidelines or codes, and a clear definition of whether amputations were the first, recurrent, or any in sequence
?	Unclear or incomplete description of reported diabetic foot disease characteristics, or using subjective measurement methods	Unclear or incomplete description of levels of amputation in line with international guidelines or codes, or unclear or incomplete definition of sequence of amputation
−	No description of diabetic foot disease characteristics	No description of levels of amputation or sequence of amputation
D	Quality of information flow	
+	Information flow was complete for each stage of the study Are the number of individuals at each stage of study reported—including number potentially eligible, number included, and number completed to follow-up?	
?	Only national health statistics used, missing information for some stages or relevant time points	
−	Poor or no description given on each stage and missing all important information	
E	Quality of study design	
+	Prospective study with diabetic foot disease or amputation as a predefined primary or secondary outcome	
?	Retrospective study with diabetic foot disease or amputation as a primary outcome	
−	Retrospective study with diabetic foot disease or amputation as a secondary outcome	

As grey literature is not peer-reviewed, an additional quality assessment of the grey literature will be performed, using the AACODS (Authority, Accuracy, Coverage, Objectivity, Date, Significance) checklist [25].

### Data synthesis

The summary statistic used for each study will be an incidence (rate per population) or prevalence proportion (%) of diabetic foot disease or diabetes-related lower-extremity amputation. The standard error for each proportion will be calculated.

A meta-analysis will be performed if three or more publications, with data collected within 10 years of each other, reporting on the same outcome and in a similar geographical area or with similar denominator population are found, providing that quality assessment items A, B, and C are scored with a “+”. Meta-analysis will be used to calculate pooled incidence or prevalence estimates using a random-effects model. Random effects will be used to give an average estimate across heterogeneous studies weighted on total sample size. The *I* ^2^ test will be used to examine heterogeneity across studies included in meta-analysis.

No meta-bias or confidence in the cumulative evidence will be calculated or assessed. Availability of prospectively registered protocols of epidemiological studies is unlikely, and as such unpublished results to calculate publication bias cannot be investigated or estimated. Further, this systematic review does not concern intervention trials, rendering the Grading of Recommendations Assessment, Development and Evaluation system not applicable. The confidence in the cumulative evidence will be a descriptive analysis based on the separate quality assessment outcomes of the included studies.

## Discussion

Epidemiological data are the backbone of research and clinical practice. However, epidemiological data are prone to various forms of bias and misinterpretation. The various intricacies of diabetic foot disease epidemiology, and their pros and cons for use in the assessment of the quality of care, have been frequently described [24, 26, 27]. However, a systematic review of the various sources of diabetic foot epidemiological data in which these intricacies are adequately taken into account and discussed is missing for the Australian situation. This may lead to the various stakeholders in this field (e.g., clinicians, researchers, politicians, and industry) misinterpreting the data, and either over- or understating the actual burden. The results of this systematic review can be used to adequately inform stakeholders in the field of diabetic foot disease and to help guide appropriate use of resources to reduce the burden of this debilitating disease.

## Additional file

Additional file 1: Appendix 1. PRISMA-P 2015 Checklist. (PDF 199 kb)

## Abbreviations

EMBASE: Excerpta Medica Database
PRISMA: Preferred Reporting Items for Systematic Review and Meta-Analyses
